# Spectral Quantification of Nonlinear Behaviour of the Nearshore Seabed and Correlations with Potential Forcings at Duck, N.C., U.S.A

**DOI:** 10.1371/journal.pone.0039196

**Published:** 2012-06-26

**Authors:** Vanesa Magar, Marc Lefranc, Rebecca B. Hoyle, Dominic E. Reeve

**Affiliations:** 1 Coastal Engineering Research Group, Marine Institute, School of Marine Science and Engineering, University of Plymouth, Plymouth, Devon, United Kingdom; 2 Laboratoire de Physique des Lasers, Atomes, Molécules (PhLAM) and Centre d’ Études et de Recherches Lasers et Applications (CERLA), UFR de Physique, Université des Sciences et Technologies de Lille, Villeneuve d’Ascq, France; 3 Department of Mathematics, University of Surrey, Guildford, Surrey, United Kingdom; University of Vigo, Spain

## Abstract

Local bathymetric, quasi-periodic patterns of oscillation are identified from 26 years of monthly profile surveys taken at two shore-perpendicular transects at Duck, North Carolina, USA. The data cover both the swash and surf zones. Singular Spectrum Analysis (SSA) and Multi-channel Singular Spectrum analysis (MSSA) methods are applied, on the shoreface, to three potential forcings: the monthly wave heights, the monthly mean water levels and the large scale atmospheric index known as the North Atlantic Oscillation. The patterns within these forcings are compared to the local bathymetric patterns; it is found that the patterns extracted using SSA and MSSA agree well with previous patterns identified using wavelets and confirm the highly non-stationary behaviour of beach levels at Duck. This is followed by analysis of potential correlations between the local bathymetry (at the two transects) and hydrodynamic and atmospheric patterns. The study is then extended to all measured bathymetric profiles, covering an area of 1100 m (alongshore) by 440 m (cross-shore). MSSA showed no collective inter-annual patterns of oscillations present in the bathymetry and the three potential forcings. Annual and semi-annual cycles within the bathymetry are found to be strongly correlated with the monthly wave height, in agreement with the SSA findings. Other collective intra-annual cycles besides the semi-annual were identified; they were all correlated with the North Atlantic Oscillation.

## Introduction

Understanding the long-term evolution of beaches over yearly to decadal time scales poses an important challenge to researchers. It is unclear how the mechanisms which force the morphology, such as hydrodynamic and sediment transport processes, affect beaches at such time scales [1], [2]. The problem is compounded by the scarcity of datasets over these periods. With the threat posed by climate change, improved understanding and better predictive tools are crucial, both for researchers and end-users alike.

**Figure 1:**
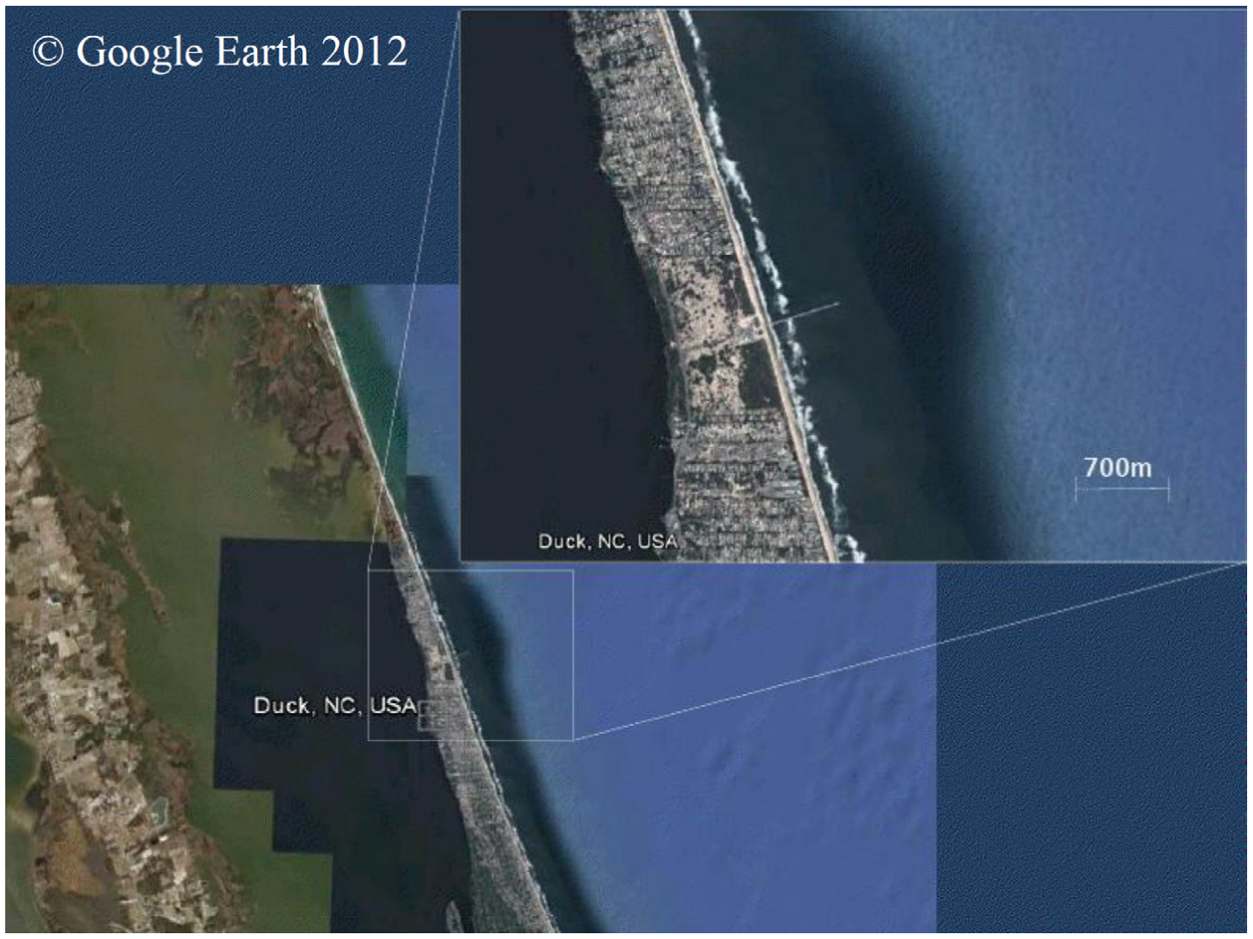
Aerial image of Duck site and close-up showing pier.

Duck, the site used in this study (see Fig. 1), consists of an open straight beach characterized by relatively regular, shore-parallel contours with a barred surf zone and a moderate slope. These characteristics change close to a pier that extends from the dune to a nominal water depth of approximately 7 m. The site has been comprehensively described by previous authors [3], [4], [5], [6] and has been the subject of numerous other studies.

This study concerns the 'coastal morphodynamics' at Duck – a term coined to describe the investigation of changes in the shape (or morphology) of coastal features such as shorelines, beaches and sandbanks; some morphodynamic studies also involve the interactions of the seabed and the hydrodynamic processes. There are several modelling approaches adopted in coastal morphodynamics: sequential models, equilibrium models, or models that are equation-based or data-based. Data-based approaches tend to be preferred in long-term morphodynamic modelling because of the difficulties in assessing how different processes affect the dynamics over extended periods [7]. Equilibrium-based models of sandbar systems assume sandbar migration towards a location that is dependent upon wave height equilibrium [8]. Sequential models work in one of two ways. In the first, single cross-shore profiles are used to analyse cross-shore sediment transport, in particular for the characterisation of cyclic patterns. The second consists of identifying beach states that characterise morphological sequences, helping to understand and classify near-shore bars in terms of their shape and dynamics [9], [10]. An important aspect of these dynamics is the observed interactions between sandbars, such as those studied at Duck [11]. Significant non-linear behaviour of inner bars was observed when outer bars were present.

There are two signal selection criteria used to analyse near-shore morphodynamics: 1) analysing spatial patterns, for instance the evolution of the sandbar crests, troughs and the shoreline, and 2) finding patterns that optimize a statistical measure, for instance patterns identified with Empirical Orthogonal Functions (EOFs), Canonical Correlation Analysis (CCA) or wavelet analysis. The first approach has been extremely popular and has contributed significantly to the understanding of sandbar dynamics [10], [11], [12], [8], [13], [14], [15], [16]. These studies focus on descriptions of bar migration, bar amplitude evolution and development of bar asymmetry. Using an optimal statistical measure as a pattern identification criterion has also been very popular; these methods are particularly useful for temporal or spatio-temporal pattern analysis. For instance, EOFs identify the linear modes of maximal variance, while CCA leads to linear modes that maximize the correlations between two time series. CCA has recently been used at Duck to analyse correlations between wave climate and bathymetric evolution, with reasonable success [17], [18]. On the other hand, wavelets have been used to analyse dominant temporal patterns of behaviour at a cross-shore transect in Duck [19]. In some cases, it has been possible to link the spatial patterns to the patterns optimizing a statistical measure [11], [12]. In other cases, the interpretation of the patterns optimizing a statistical measure may be less obvious; for instance, the origin of the 1–2 yearly temporal seabed pattern that have been observed Duck is unknown [19], so additional assessments are still needed.

We postulate that different bathymetric regions have different non-linear behaviour depending on their cross-shore and alongshore location. Several of the observed patterns are also well correlated with patterns embedded within potential forcing mechanisms such as monthly wave patterns, monthly sea level patterns or the North Atlantic Oscillation (NAO) index (herewith simply called the NAO). The NAO is a climatic fluctuation of atmospheric pressure at sea level between Iceland and the Azores [20]. To analyse the patterns we use SSA and MSSA, two data-based, non-linear methods that optimize modes of maximal variance. The methods are applied to time-lagged copies of bathymetric (and hydrodynamic or atmospheric index) time series. They were chosen because they are good at identifying underlying temporal and spatio-temporal patterns of oscillation, even for short and noisy data, as is the case for most coastal morphodynamics datasets [21]. Non-linear patterns of the behaviour of bed-levels at Duck are quantified using SSA and spectral density analysis (SDA) to characterise quasi-periodic oscillations. Possible correlations between these patterns and those embedded within large-scale phenomena will be discussed, as they might potentially be the origin of the observed behaviour. Correlations between them will be confirmed by further multivariate EOF (MEOF) and multichannel singular spectrum analysis (MSSA) studies.

It has been shown, for example, that SSA is capable of describing the dynamics of paleoclimatic oscillations obtained from marine sediment core measurements that had as few as 184 data points, as well as capable of identifying the statistically significant number of degrees of freedom [21]. Another way of identifying the statistically significant degrees of freedom is by computing the correlation dimension. However, it has been shown that reliable correlation dimension computations, for the same core measurements, were not possible due to the data’s shortness and noisiness [21], [22]. Indeed, it is well known that, in general, the number of data points needed for accurate computation of the correlation dimension would be much larger - more likely on the order of one thousand or larger [23], [24]. When analysing temporal patterns, however, it is also important for the frequency of sampling to be significantly larger than the frequency of the studied pattern, regardless of the size of the dataset [25]. For our purposes SSA (and by extension MSSA) is a perfectly suitable technique since our interest focuses on the quasi-oscillations underlying the dynamics and these oscillations are well captured by such techniques.

SSA has some similarities with EOFs, first applied to analysing beach variations by [26]. While the implementation of EOF is relatively straightforward, with SSA intermediate computations and tests are necessary to obtain a set of uncorrelated reconstructed components and avoid misinterpretations. However, SSA is superior to EOF in that it can identify non-linear, temporal patterns underlying the dynamics. Both SSA and EOF, as well as MSSA and Complex EOF (CEOF), have very recently been exploited to analyse long-term sea level changes [27], as well as behavioural patterns in coastal features such as shorelines [1], [28], [29], [30], and nearshore sandbars [12], [31]. One major benefit of SSA is its ability to capture the non-stationarity in the patterns, like wavelet analysis and unlike spectral analysis [32], [33].

SSA was used to identify the long-term trend of the shoreline at Duck between 1980 and 1993 [1]. The component of the signal resolved by the first three eigenvalues was analyzed. Their reconstruction showed no evidence of quasi-periodic oscillations of the shoreline. Rather, it led to the identification of an accretional trend from 1981 to 1990, followed by significant erosion from 1990 onwards. This contrasted with their findings at Ogata beach, Japan, where a clear linear trend of erosion, with a 5-yearly cycle superimposed, could be identified.

Crucial in SSA is the specification of signal and noise, as demonstrated by a study of shoreline evolution at Lubiatowo, Poland [28]. In this study three long-term patterns were identified, with periods of 9, 16 or 32 years, at different positions along the coastline. Inter-annual cycles of 2–3 and 3–4 years were also found. However, significant variability also occurred at some positions. The study also pointed to the possible existence of chaotic behaviour, as resolved by the second eigenvalue, which the authors linked to a response to extreme events. An association between the rhythmicity of the bed-level variations and sand bar propagation was also postulated, supported by the existence of high correlations between shoreline positions and the inner bar crests [34]. The authors also explain some findings in terms of self-organisation.

MSSA follows the same principles as SSA, but is performed on a vector that contains variations at different positions, rather than at a single location, making it more likely to identify collective patterns of behaviour. MSSA is sometimes called multivariate extended empirical orthogonal function (MEEOF) analysis [35].

MSSA has been applied at Lubiatowo to identify the collective patterns of behaviour of the shoreline [29]. Three collective quasi-periodic oscillations were identified with 7–8 year, 20 year and several decade-long cycles. Of interest is the fact that the collective patterns have different periodicities than the local bathymetric patterns. This may be explained by two observations. First, the collective patterns will necessarily appear in the SSA at some individual positions, but not everywhere as the dominant patterns of behaviour. Second, because the data only extends for 16 years, there is necessarily some uncertainty on the exact periodicity of patterns with periods above 5 years, so the 7–8 local pattern and 9-year collective pattern may actually correspond to the same pattern. The same would hold for the 16-year local pattern and the 20-year collective pattern. The authors commented that the shortest cycle might be forced by the North Atlantic Oscillation (NAO), which has an important influence in the Baltic Sea. Evidence supporting that the NAO may affect the bathymetry through a gentle coupling with the hydrodynamics and the seabed has been found recently [36].

The spatial regularity of near-shore patterns implies that the underlying dynamics may be understood and, most importantly, described using simple physical mechanisms [37]. Looking for mechanisms acting in the long term is particularly important. Within the surf zone and at the shore, the occurrence of spatially rhythmic patterns has been attributed to the presence of low-frequency waves, such as infra-gravity waves [38], [39], [40]. However, infra-gravity waves are only responsible for the short-term response of some beaches, not the observed inter-annual behaviour. At such large timescales, the wave-related parameter that is of importance is the monthly averaged wave height. Note that we are not implying a simple relationship between the waves and the bathymetry, whose response to forcings has been shown to be non-linear in other studies [37]. This non-linearity could be reflected in the difference between amplitudes and phases, as well as in the characteristics of the regime changes observed in the corresponding time series. In the case of alongshore parallel bars, the bar crests and troughs may cause redistribution of wave energy, variations in the wave breaking point and wave transformation processes (i.e. wave refraction, reflection and diffraction). This produces a radiation stress which is no longer in equilibrium with the set-up and set-down, creating a steady circulation [37]. Aside from the monthly averaged wave heights and atmospheric phenomena such as NAO, it is likely that tides and storm surges also have an effect on long-term morphodynamics. Tides are fully predictable, shallow-water waves generated by gravitational forces between the Earth, the Sun and the Moon. Storm surges change the water depth through pressure forces, wind stresses and wave set-up and set-down, in turn changing the water depth at the bar crests and troughs and creating the radiation stresses mentioned above. Given that tides and storm surges may be combined in a single parameter, namely the mean water level, some of the patterns observed in the bathymetric evolution may potentially be correlated with patterns found within a water level time series.

In Sect. 2, below, the case study data is presented and the SSA and SDA techniques are explained in more detail, together with the MEOF and the MSSA. The results are presented in Sect. 3 and discussed in Sect. 4. Sensitivity to window length changes is discussed in Sect. 4.1. Comparisons with previous investigations are presented in Sect. 4.2. In Sect. 4.3, SSA and SDA of the monthly averaged wave heights at a near-shore wave gauge are performed and the patterns are compared to those of the bathymetry, together with similar analyses for the North Atlantic Oscillation (taken as an average over the whole area of study). This is to illustrate the possible correlations with large-scale phenomena. Section 4.4 discusses linear correlations between the full set of bathymetric measurements and the monthly MWH, monthly mean sea level and monthly NAO. Coherent spatio-temporal correlations identified via MSSA are also discussed. Finally, Sect. 4.5 contains a summary and some concluding remarks. Websites are referred to as data or software sources, when appropriate, in accordance with copyright requirements.

## Methods

### 2.1 The Source and Nature of the Data under Analysis

Data were provided by the Field Research Facility (FRF), Field Data Collections and Analysis Branch, US Army Corps of Engineers (USACE), Duck, N. C., available at http://www.frf.usace.army.mil. The data show measurements taken at Duck since June 1981, on a monthly basis, over a series of 26 shore-perpendicular transects, generally extending from the dune to approximately 950 m offshore. Four lines, located at approximately 500 m and 600 m North and South of the pier, have been surveyed every fortnight. Table 1 shows the average alongshore locations of these four lines, with their associated measurement errors, as well as their transect number (FRF location code). The alongshore coordinate is assumed to be *y* and the cross-shore coordinate is *x*. The baseline is a shore-parallel line with its origin at the South-East corner of the FRF property. On-going quality control and early publication of the surveys for these transects led to a significant number of studies based on them (see Introduction). Moreover, the bathymetric evolution at these profiles is the least affected by the presence of the pier [6]. However, important differences in the behaviour North and South of the pier, in particular regarding the sandbar system, have been observed [41].

**Table 1:** Correspondence between transect number and transect alongshore position for the four best surveyed transects at Duck, North Carolina, USA.

TN	190	188	62	58
*Y*	−91	0	1008	1097
Δ*y*	32	17	98	91

TN is the transect number, the averaged alongshore position Δy the absolute error around this average.

Duck is an example of a net offshore bar migration (NOM) system or a system with inter-annual cyclic sandbar behaviour [41], [42]. NOM is a cyclic phenomenon consisting of three stages: bar generation near the shoreline; systematic offshore migration of the bar across the surf zone during high energy periods; and disappearance of the bar in the outer surf zone [43]. Sandbar formation and migration may also be triggered by previous profile geometries, with the degeneration of an outer bar often leading to formation of a new bar near the shoreline [42]. NOM systems are often alongshore- coherent, but at Duck this coherence is somewhat broken by the presence of the pier. Profiles at Duck vary from unbarred to triple- barred, with the most common profile configuration being a double-bar system consisting of a narrow inner bar and a broad outer bar [3], [4], [5].

Three parameters may characterise a NOM cycle at each stage: the average cross-shore distance over which bars migrate; the average duration of the bar migration; and the average return period of migration cycles. These parameters are site-specific, and at Duck they vary North and South of the pier. While the total distance and the total duration of bar migration are relatively similar at Duck North (DN) and Duck South (DS), the average return period is significantly different. The total distances travelled by the bars are 289.6 and 289.1 metres; their total duration over all three stages is 4.4 and 4.1 years at DS and DN, respectively. However, for the second, most active stage, the return period at DS is 3.2 years while at DN it is 6.8 years. The sand is transported back to the shoreline during fair-weather periods through a variety of mechanisms: transport in the outer surf zone is driven by wave asymmetry, while in the inner surf zone it also occurs through migrating bodies such as transverse bars or bar bifurcations generated during bar splitting processes [5], [44], [45], [46]. The wave climate has a very strong seasonal pattern, with storms occurring mostly during the winter. Since storms cause sandbars to move offshore and bars move shore wards during milder weather conditions, annual patterns are expected to appear within the bathymetry.

In this study, the two outermost transects, transects 58 (DN) and 190 (DS), were first chosen to analyse the local quasi-oscillations embedded in the most active part of the surf zone. This reduced the cross-shore range to 390 m, between *x* = 100 m and *x* = 490 m, in agreement with the range analysed in other studies [47]. It also includes most of the NOM width, that is, the region of sandbar migration [5]. However, the study area was extended for linear correlation and MSSA analyses. It was extended to *x* = 80 m to include the shoreline processes in more detail, and to *x* = 520 m to check the type and strength of the correlations further offshore. A spatial and temporal Akima interpolation was performed on the data to obtain depth values at constant 10-m intervals and at even time-steps of 30 days [48]. These time and space interpolation intervals prevent over-interpolation of the data. This procedure led to a 299-point time series at every bathymetric position, spanning from July 1981 to January 2006. Cross-shore bathymetric anomalies, with respect to the time-average at each position along the transect, are shown in panels 2A and 2D for transects 58 and 190, respectively.

### 2.2 Methods Used for Local Pattern Analysis

Two complementary methods were used to identify local patterns of behaviour: SSA and SDA. Both methods are described in detail in Sect. S1.1 in Appendix S1; thus, we only present a brief summary of SSA. Essentially, SSA is a non-linear eigenvalue problem for an M-dimensional sequence *Z_n_ = {z_n_,z_n+1_,…,z_N+M-1_}*, for *n* = 1,2,…,*N*−*M*+1, of time-lagged copies of the bed level *z*(*t*), at times *t = iτ_s_*, *i* = 1,2,…,*N. N* is the length of the time series, *τ_s_* the sampling interval and *M*<*N* is the window length. Time scales of the dynamics that can be reconstructed from this time series are between *τ_s_* and the window time span. Reconstructed components (RCs) linked to the eigenvalues of *Z_n_* have the same time span as the original time series and isolate one or more of the quasi-oscillatory patterns. This depends on the number of eigenvalues used for the reconstruction. In this way, the signal was separated into a trend and a detrended signal using a window length, *M*, of 4 years. The choice of *M* is arbitrary, and in this particular study it does not need to be chosen with care because all the patterns (both the short and long-period oscillations) are being analysed. The trend corresponds to linear variations, as well as to patterns with periods longer than 3–5 years, and is the RC of the first few eigenvalues of the SSA with *M* = 4. Long-period oscillations (LPOs), embedded within the trend signal, can be analysed with an SSA window length of 9 years. Conversely, the detrended signal contains short-period oscillations (SPOs) with periodicities of less than 5 years. These SPO periodicities can be determined with a further SSA on the detrended part of the signal. The overlap between LPOs and SPOs is due to the chosen window length of 4 years for the initial SSA. The robustness of the observations to small changes in the window length will be discussed in more detail later.

Patterns embedded within the MWH, MWL and monthly NAO were also extracted following the same procedure. The patterns obtained were compared to those within the bathymetry to identify locations where similar patterns are observed. This led to an evaluation of correlations between bathymetric and hydrodynamic/atmospheric patterns. Note that SSA would give a non-linear measure of the correlations. Linear correlations were found as a preliminary step in the analysis of spatio-temporal patterns of behaviour.

### 2.3 Correlation Analysis for Collective Patterns of Behaviour

Spatio-temporal collective bed-level patterns were analysed for all bathymetric surveys between transects 58 and 190, inclusive. A linear correlation analysis was first performed by computing the correlation coefficient matrix, *r*, between the bed-level time series and that of each of the potential forcings. This led to three different correlation coefficient matrices: one for the correlations of the bathymetry with the NAO; one for correlations with the MWH; and one for correlations with the MWL. The values in *r* were used to produce correlation coefficient contours to identify locations where the correlation between the bed-levels and the forcing is strongest. The analysis is intrinsically linked with the MEOF analysis carried out as a preliminary step for MSSA for all the bathymetry transects. This is because the correlation coefficient is equivalent to the covariance matrix, *C*, normalised by the square root of the product of the covariance at the associated diagonal elements [49]. MEOF is also based on the covariance of the multivariate matrix. The MEOF method is described in detail in Sect. S1.2 in Appendix S1.

**Figure 2:**
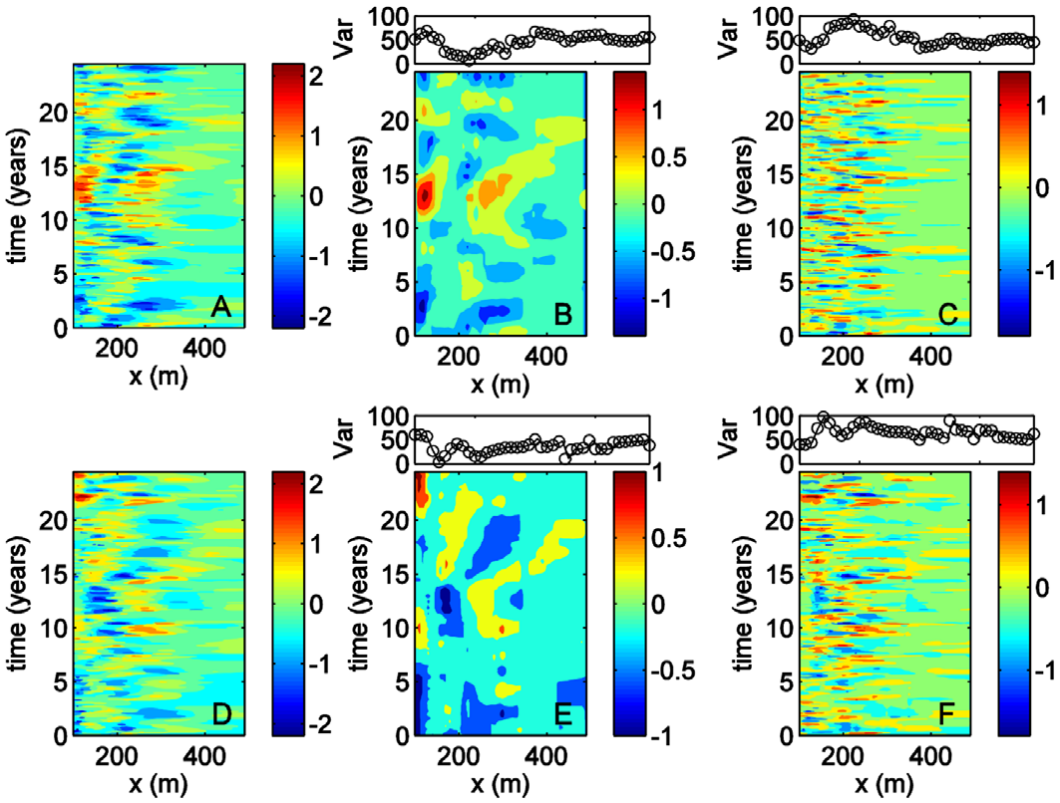
Data, trends and detrended signal contours for transects 58 and 190. The beach elevation (in metres) is displayed in panels A and D, the trend signal elevation (in metres) in panels B and E and the detrended signal elevation (in meters) in panels C and F. The upper sub-panels in panels B, C, E and F show the percentage of variance resolved by the corresponding signal.

MEOF reduces the number of channels, from 1128 to 3, in a process called pre-filtering. This corresponds to the MEOF RCs in directions of maximal variance of the potential forcings. EOF1 corresponds to the NAO, EOF2 to the mean wave heights (MWH) and EOF3 to the mean water levels (MWL). The system of three channels is then analysed with MSSA to identify which collective, bed-level spatio-temporal patterns may be correlated with NAO, MWH or MWL. The MSSA technique is described in Sect. S1.3 in Appendix S1.

## Results

### 3.1 Identification of Trend and Detrended Signals

The trends, shown in Figs. 2B and 2E for transects 58 and 190, respectively, capture the dominant patterns observed in Figs. 2A and 2D well. These seem to correspond to the extrema in the bathymetry and therefore are likely to correspond to the sandbars’ extrema. This is supported by the fact that some of the travelling characteristics of the sandbars have also been captured; the extrema, as well as surrounding bathymetric contours, appear to travel offshore as time progresses.

For transect 58, the patterns extracted resolve approximately 50% of the variance in regions where the extrema are well resolved. At other locations, such as between 130 and 220 m offshore, the variance resolved by the trend is slightly less than 50%. However, these results indicate that the trend and detrended signal are equally important because they resolve an equal proportion of the total variance; hence, both should be analysed further.

The variance for transect 190, resolved by the trend and the detrended signals, is about the same. However, with the trend accounting for slightly less than 50% of the variance in this case, both signals need to be analysed. As with transect 58, the detrended signal, which contains the higher frequency oscillations, with periods generally shorter than 4 years, seems to resolve slightly more of the variance between 130 and 220 m offshore.

**Table 2:** Periods of dominant oscillatory patterns at selected locations along transects 58 and 190, with the uncertainty or confidence interval in brackets.

	Transect 58		Transect 190	
x (m)	LPOs (yrs)	SPOs (yrs)	LPOs (yrs)	SPOs (yrs)
120	6.5(2)	1(0.7)	15.5(1.5)	1(0.1),2.6(0.1)
130	**16.4(4)**	1(0.1)	**8(1)**	1(0.1),2.7(0.2)
140	**11.4(5.1)**	1(0.2)	**1.8(0.2),** **3.1(0.5)**	1(0.1),2.4(0.2)
			**5.6(0.4)**	
150	3.1(0.6)	1(0.1)	**6.5(0.5)**	2.1(0.2),1(0.1)
180	3.9(0.3)	1(0.1)	**13(1),** **1.7(0.1)**	2.4(0.2),0.9(0.2)
190	4.7(0.8)	1(0.1)	1.7(0.3)	2.2(0.2),1(0.1)
200	6.4(0.6)	all below 1	5.5(0.2)	2.9(0.5),1.4(0.2),1(0.1)
220	**7.9(2.4)**	0.8(0.1),1.8(0.1)	3.6(0.4)	4.4(0.6),1.4(0.2),1(0.1)
230	**6.3(1.2)**	2.2(0.5)	**10(1)**	1.5(0.1),1(0.1)
240	**4.4(0.8)**	1(0.1),1.9(0.2)	**12(1)**	2.9(0.5)
250	**5.4(0.9)**	1(0.2),1.8(0.1)	12(1)	2.6(0.3),1.6(0.3),1(0.2)
260	**5.2(0.7)**	1.8(0.2),4.2(0.6)	14(2)	2.5(0.3),1.6(0.3),1.2(0.2)
310	5.8(0.7)	1.1(0.1),1.7(0.3)	**5.8(0.2)**	1.2(0.2),1.6(0.3),2.5(0.3)
340	**7.3(1.8)**	1.5(0.3)	**6.5(0.5)**	3.1(0.4),1.5(0.3),1(0.2)
350	**9.8(4)**	2.1(0.9)	**6.5(0.5),** **8(0.4)**	1(0.2),1.5(0.2),2.2(0.1)
370	3.4(0.5)	1.4(0.1),4.8(1.1)	**6.2(0.4),** **9.3(0.6)**	1(0.5),2.2(0.2)
390	3.4(0.2)	2.2(0.6)	5.8(0.4)	1(0.5),1.6(0.5),2.3(0.2)
410	3.3(0.9)	1.4(0.1)	4.3(0.2)	1(0.1),2(0.2),3.2(0.6)
470	6.7(0.8)	2.5(0.9)	3.2(0.2),4.8(0.2)	same as 410
480	10.3(6.2)	2.6(0.6),3.3(0.8)	3.3(0.2)	same as 410
490	7.1(2)	2.5(0.5)	4.8(0.2)	1(0.1),1.3(0.4),3.4(0.6)

In bold are locations where the patterns resolve most of the variance within the data.

**Figure 3:**
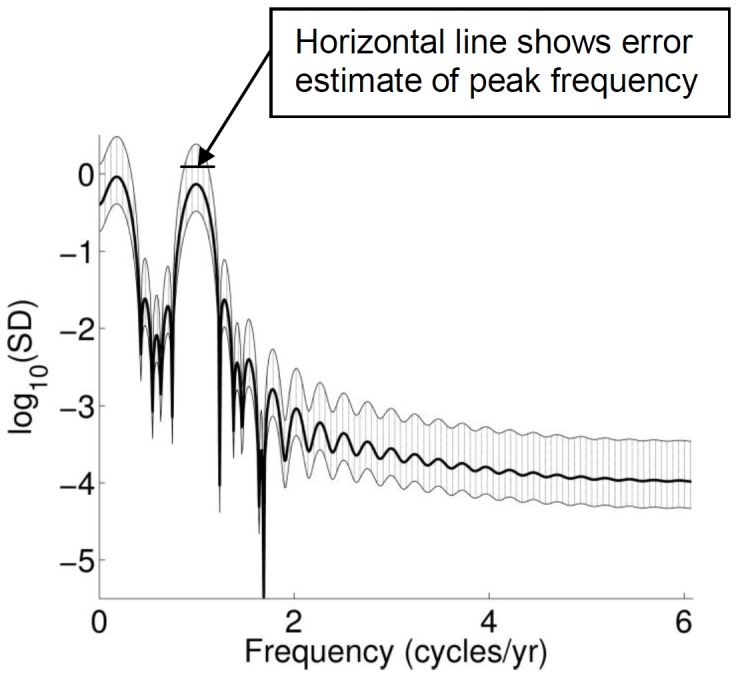
Spectral density plot for the reconstructed component of the SPOs at x = 160 m for transect 58. The thin lines indicate the 95% confidence interval for the spectral estimates. The horizontal line shows the error of the peak frequency estimate for the quasi-yearly cycle.

The periods of the underlying cycle were computed all along transects 58 and 190; Table 2 shows the results at selected locations along the profiles. The uncertainties in the periods were estimated from figures of the power spectrum, as well as from the uncertainty of the power values at each frequency. An example of a power spectrum is shown in Fig. 3, corresponding to position *x* = 160 m at transect 58. If the uncertainty interval of the power value extends to, or beyond, the peak power value, then the uncertainty of the position of the peak also extends to the frequency at that power value. Figure 3 complements Table 2, which shows the uncertainties for the cycle periods in square brackets.

### 3.2 Identification of Underlying Quasi-oscillations

**Figure 4:**
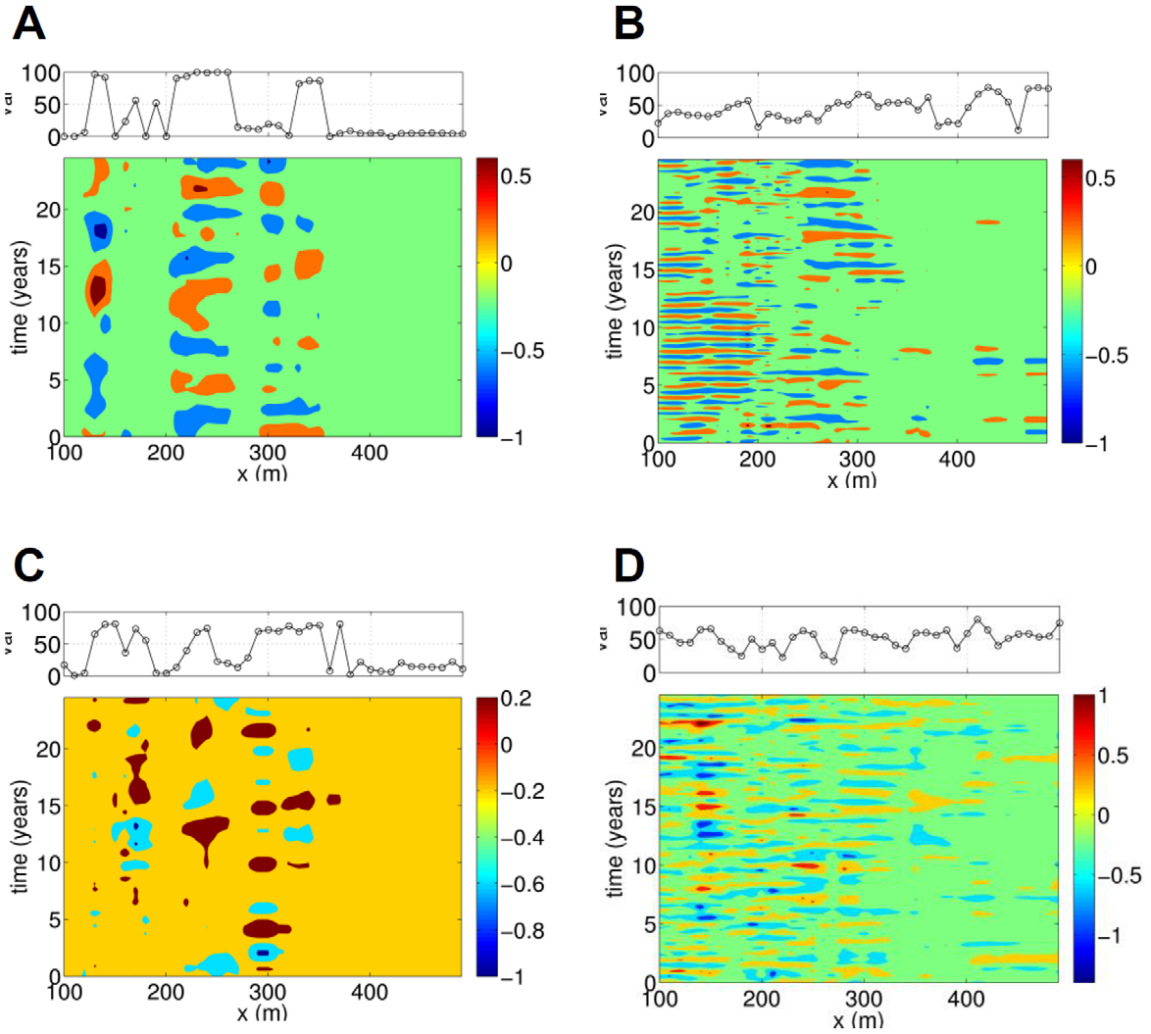
LPOs and SPOs and their resolved variance (top) for transects 58 (LPOs shown in A; SPOs in B) and 190 (LPOs shown in C; SPOs in D).

Long period oscillations (LPOs) and short period oscillations (SPOs) - with periods above and below 4 years, respectively - were then extracted from the trend and the detrended time series. The RCs obtained when combining all of the LPOs and SPOs in transect 58 are shown in Figs. 4A and 4B, respectively. For transect 190, the LPOs and SPOs are shown in Figs. 4C and 4D.

In relation to the LPOs, it is clear that these patterns have an important contribution below 350 m to 400 m offshore; the variance resolved by these patterns - shown in the upper plots of Figs. 3 - typically takes large values there. At both transects the signal contains roughly three regions where quasi-oscillations are important. At transect 58, these regions extend between *x* = 130 and 140 m, *x* = 210 and 260 m and *x* = 340 and 350 m; at transect 190, they extend between *x* = 130 and 180 m, *x* = 230 and 240 m and *x* = 290 to 370 m (see pattern cycles in bold font in Table 2). Hence, there is agreement in the number of regions but their extent differs. The main periods of the quasi-oscillatory patterns are summarised in Table 2, which shows that there is a lot of variability. However, between 220 m and 370 m offshore, the periods of the patterns are close to those found within the NAO (see the end of this subsection).

The SPOs, in contrast, may have an important contribution throughout the region under consideration, as the variance they resolve is usually around 50% - see upper plots in Figs. 4B and 4D. Based on the information in Fig. 4B and Table 2, the transect 58 signal contains a very clear yearly cycle between *x* = 100 m and *x* = 300 m. A 1–2-year cycle is also present between 200 m and 300 m offshore. The SPOs have periods between 1.1 and 1.3 years around *x* = 310, and between 2.2 and 2.5 years further offshore. Beyond 300 m, the amplitudes of the patterns are very small, possibly because at these depths the effects of the mechanisms driving such patterns are also smaller. Examination of Fig. 4D and Table 2 shows that the yearly cycle is present throughout transect 190, together with a cycle whose period ranges between two and three years. There is sometimes a third cycle, from 200 m to 490 m, with a 1–2-year period. The periods of the SPOs are close to those found within MWH, MWL and NAO, as we will discuss in subsequent sections.

It is noteworthy that, between 350 m and 490 m offshore, the periods of the dominant patterns may vary from one to 10.3 years at both transects. Inter-annual patterns of variable periods were also found closer to the shore. This suggests that differences in the temporal patterns of the sandbar life cycle between different sides of the pier are not reflected in differences of the seabed elevation variations between those sides.

Finally, at some locations, the detrending seems not to have worked properly. This is the case, for instance, between 370 and 390 m for transect 58, and at 140 and 200 m for transect 190. In all cases the patterns have periodicities close to 4 years and appear to have been misclassified within the trend or detrended signal. In some cases the uncertainty interval includes the 4-year window length size even though the value of the peak lies incorrectly in the trend or detrended signal. In other cases, it is the lack of a pattern of a period longer than 4 years which leads to the identification of patterns between 3 and 4 years within the trend. Such issues are related to some limitations of SSA in unequivocally identifying patterns which have periodicities close to the size of the window length.

Patterns embedded within MWH, MWL and the monthly NAO were also extracted following the same procedure and compared to those within the bathymetry to identify locations where similar patterns are observed.

**Figure 5:**
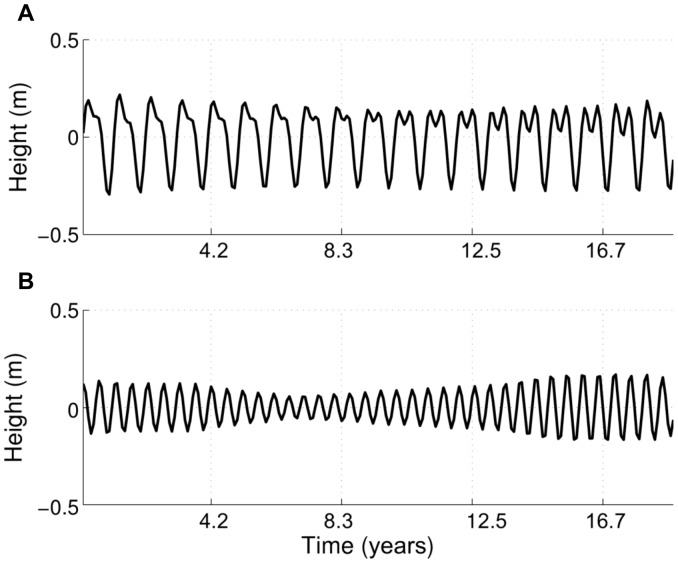
SSA reconstruction of wave dynamics. The first fundamental pair is shown in A and the second one in B.

It is expected that a yearly cycle will be embedded within the wave data; hence, it may potentially be correlated with the bathymetric yearly cycle. This was investigated using wave data from a wave gauge array consisting of 15 pressure gauges mounted 0.5 m off the sea floor at a depth of around 8 meters. The array is located between 835 m and 955 m offshore and between 735 m and 990 m alongshore, i.e., in the vicinity of transect 58. At this location the mean wave period is around 9s, with a standard deviation of 0.76s; there is little variation in the monthly averaged wave period. Therefore, the only wave parameter showing significant variation at this location is the wave height. Since the bathymetric analysis is performed on monthly interpolations of the data, and our interest lies in the long-term (yearly to decadal) evolution of the seabed, we deem it sufficient to consider the monthly averaged wave heights at this location. This is equivalent to utilising appropriate means in the wave forcings in relation to the timescales of the studied bathymetric change [50]. SSA of the monthly averaged wave heights clearly indicates the two main underlying quasi-oscillations are predominantly annual and semi-annual, respectively (see Fig. 5).

As mentioned in the Introduction, storm surges have been shown to have an important influence on coastal erosion. Although the variation of water depth throughout the bathymetry is unknown, a measure of the local water depth, or the mean water level, MWL, is given by tidal gauge measurements at the site, collected by NOAA. An SSA of the MWL highlights two inter-annual oscillations of less than 9 years, with periods of 5.7–6.2 years and 1.89–2 years. Four intra-annual patterns are also identified, with periods of 2.47–2.53 months, 3.9–4.1 months, 5.8–6.1 months and 1 year.

To check which patterns are embedded within the NAO time series, NAO index data were obtained from the coastal research unit of the University of East Anglia (see http://www.cru.uea.ac.uk/timo/datapages/naoi.htm) and analysed with SSA. The annual NAO index between 1850 and 1999 was extracted and SSA was applied using a window length of 40–50 years. This allowed identification of inter-annual as well as some decadal patterns of oscillation underlying the dynamics. The inter-annual patterns identified were cycles of 7.1–8.8 years and 2.7–2.9 years, with a smaller contribution from cycles of periodicity between 3.8 and 5 years. A contribution with a periodicity between 12 and 13 years was also found.

Linear correlations may also be computed. This was done throughout all of the bathymetric surveys and the results are discussed next.

### 3.3 Spatial and Spatio-temporal Correlations between the Bathymetry, Waves, Mean Water Level and NAO

**Figure 6:**
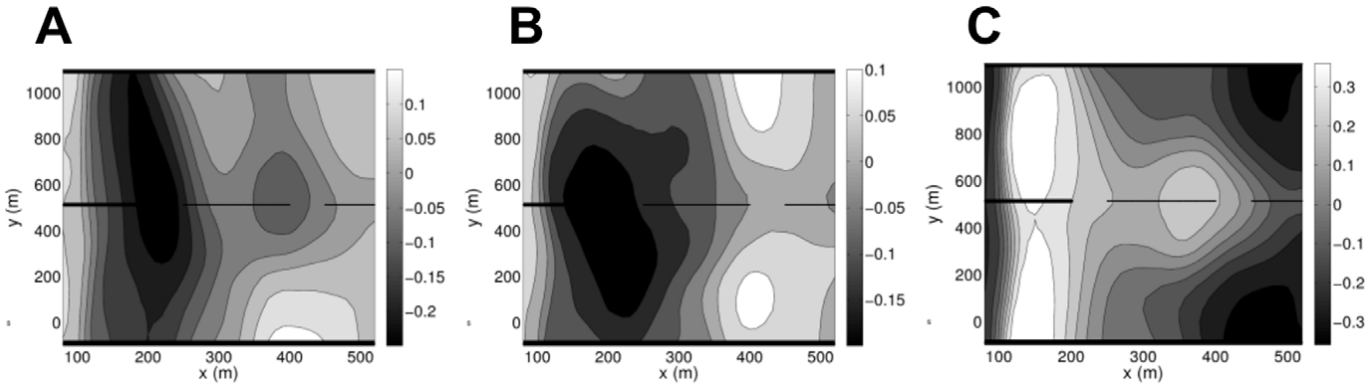
Correlation contours between the seabed and the (A) NAO, (B) the MWH and (C) the MWL, respectively. Tr. 58 (top, Northern side) and Tr. 190 (bottom, Southern side) are highlighted (solid lines). The pier is located at *y* = 513 m (dashed line).

The existence of possible correlations between the bathymetry and the NAO, MWL and MWH, and whether these phenomena have a local influence, or act throughout the whole bathymetry, are addressed in this section. These phenomena are evaluated at a single location, as explained in Sect. 2. In our analyses we used measurements of the bathymetry at several locations along each transect. Contours of the correlation coefficient, *r*, computed using a simple correlation analysis, are shown in Figs. 6 for the bathymetry and the NAO (panel 6A), the MWH (panel 6B) and the MWL (panel 6C). The locations where *r* is largest seem to agree with locations where the SSA analysis indicated such correlations between the bathymetry and the atmospheric/hydrodynamic phenomena could exist (see Sect. 4.3). The maxima and minima of *r* show locations where the correlations are strongest, independently of the magnitude of *r*.

In order to assess the overall strength of the (linear) correlation for each of the variables the following correlation criteria may be useful: a) strong, with |*r*|>0.5; b) moderate, with 0.3<|*r*|<0.5; c) weak, with 0.1< |*r*|<0.3; d) no or very weak, with |*r*| <0.1. With these criteria, the percentage correlation between bed-levels and potential forcings may be computed for all of the bathymetric profiles. For NAO: 65.6% is very weakly correlated; 34.4% is weakly correlated. For MWH: 67.6% is very weakly correlated; 32.4% is weakly correlated. For MWL: 28.3% is very weakly correlated; 50.5% is weakly correlated; 21.2% is moderately correlated. Note that the values of these correlation coefficients are very low, but they only give a measure of the linear correlation between the bathymetric evolution and the potential forcings, while SSA and MSSA give a non-linear measure of correlations.

**Table 3:** This Table is associated with the MEOF analysis and is an extract of the spatial eigenvector matrix resulting from the MEOF decomposition explained in Sect. S1.2 in Appendix S1.

EOF values with all seabed surveys				
Forcing:	EOF1	EOF2	EOF3	EOF4
NAO	0.998	−0.067	−0.012	−0.0005
MWH	0.067	0.998	0.0005	−0.0009
MWL	−0.012	0.001	−0.999	0.044
EOF values with Transects 58 and 190 only				
Forcing:	EOF1	EOF2	EOF3	EOF4
NAO	0.998	−0.067	−0.013	−0.0001
MWH	0.067	0.998	0.0004	0.003
MWL	−0.012	0.001	−0.940	−0.335

It shows the last three rows of the first four spatial eigenvectors. By construction of the multivariate matrix, these last three rows are associated with the North Atlantic Oscillation (NAO), Monthly-averaged Wave Heights (MWH), and the Monthly mean water level (MWL). The forcings have been arranged so that the largest elements in the Forcings/EOFs matrix are in the diagonal.

Table 3 shows the first four EOFs from the MEOF analysis; the rows correspond to each of the potential forcings for transects 58 and 190, and then for the full set of bathymetric measurements at locations between and including these two transects. The potential forcings have been organised so that the one at the top has the largest absolute value contribution for EOF1, the second for EOF2, and so on. It is clear that each potential forcing may be unambiguously associated with a particular EOF. EOF4 has been included to show that, for this eigenvector, all potential forcings generally have a contribution at least one order of magnitude smaller than for eigenvectors EOF1 to EOF3. The eigenvector EOF1, corresponding to the largest eigenvalue, is correlated with NAO regardless of whether considering transects 58 and 190 or all measured bathymetric profiles. Similarly, EOFs 2 and 3 are correlated with MWH and MWL, respectively, in both cases. The magnitudes of the components corresponding to potential forcings in EOFs 1 to 3 are also very close. In fact, they are equal for EOF1 and EOF2 at the level of accuracy considered here. This is true whether considering the two transects or all of the bathymetric measurements together. Hence, the potential forcings are correlated in the same way with transects 58 and 190 as they are with the full set of measurements of the bathymetry; thus, we can use the latter for subsequent analyses.

The MEOF analysis indicates that the first three EOFs are strongly linked to the three forcings considered and together they resolve 98.25% of the variance. Note how large the resolved variance is compared to the values of the correlation coefficients. This shows that even though the correlations are small, correlations with any other phenomenon would be significantly smaller; thus, we can confidently say that NAO, MWH and MWL are the only relevant potential forcings. Also, since these three EOFs resolve so much of the variance, their associated RCs are used in the MSSA as input channels instead of the original dataset. This simplifies the MSSA considerably as the number of input channels is reduced from 1128 to 3. The spatio-temporal oscillations within the filtered system (with 3 input channels rather than 1128) are then analysed with MSSA. From the MEOF results it is known that input channel 1 is linked to NAO, input channel 2 to the MWH and input channel 3 to the MWL.

The MSSA analysis will lead to a number of MSSA components. In analogy with EOF and SSA, each of the MSSA components has its corresponding eigenvalue, spatio-temporal principal component (ST-PC) and spatio-temporal EOF (ST-EOF). The criteria presented by [51] were used to identify coherent patterns within the ST-PCs. The method is described in detail in Sect. S1.3 in Appendix S1. It consists of finding pairs of consecutive eigenvalues which pass a number of tests. The pairs passing such tests correspond to a quasi-oscillatory pattern. It was found that the first 7 pairs of eigenvalues, ST-PCs and ST-EOFs could be linked to quasi-oscillatory patterns. Since most of variance was resolved by these 7 pairs the analysis was stopped at this point.

**Figure 7:**
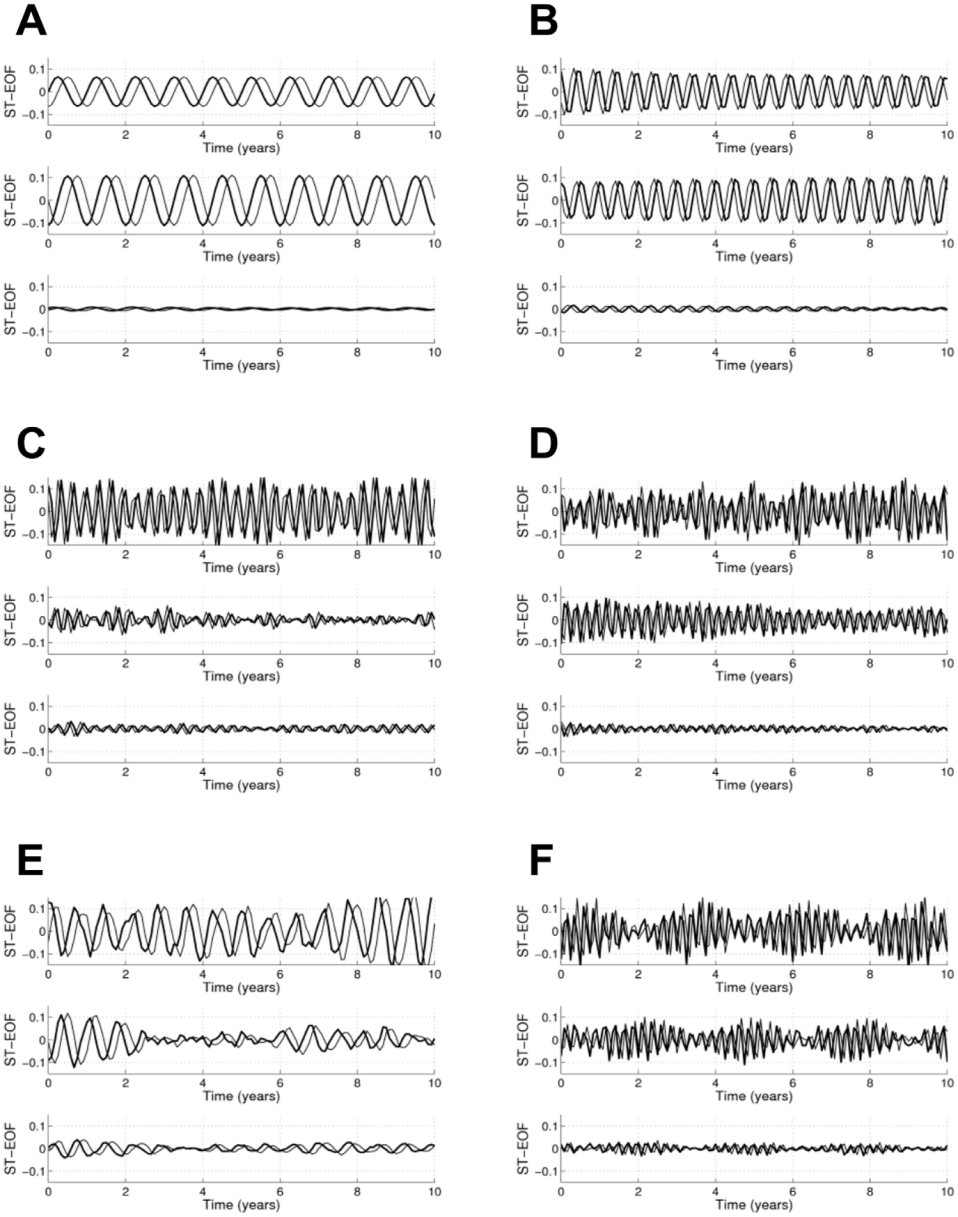
First six ST-EOFs pairs, with their associated resolved variance. The abscissa represents time (in months) and spans the window length *M* = 121 months. A: pair 1–2; B: pair 3–4; C: pair 5–6; D; pair 7–8; E: pair 9–10; F: pair 11–12.

**Figure 8:**
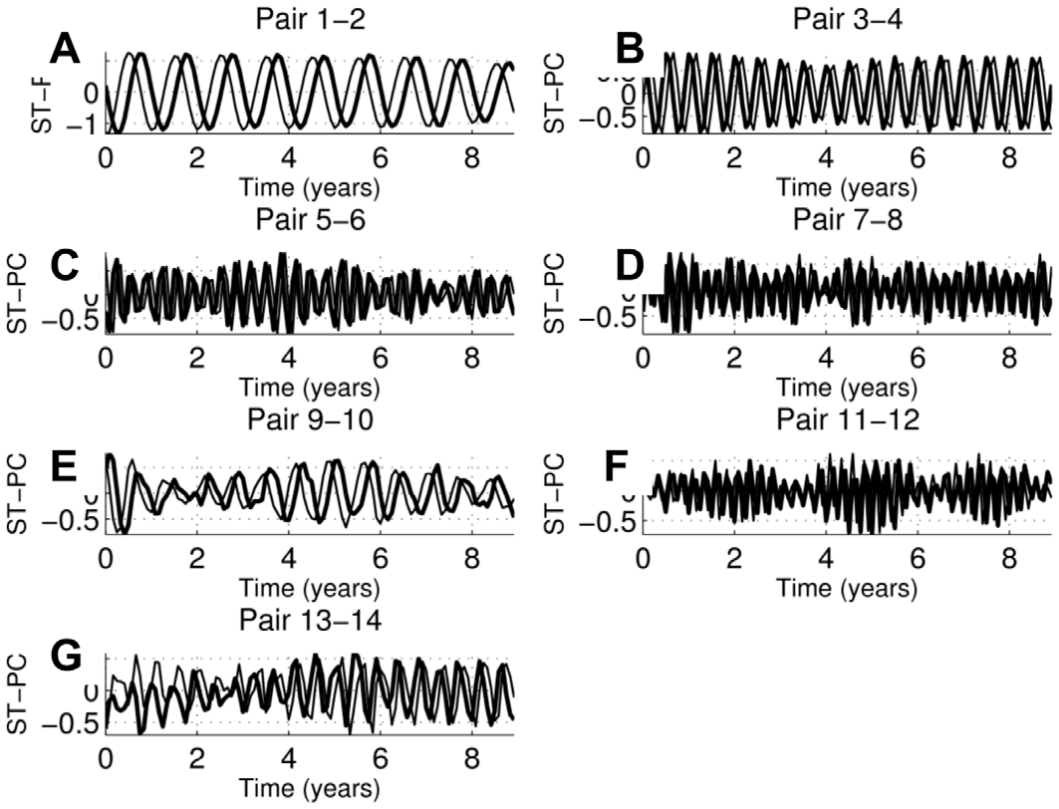
ST-PCs for consecutive MSSA components at channel 1; the ST-PCs span the window length N' = 108 months.

Once pairs of eigenvalues corresponding to embedded quasi-oscillations are identified, the periods of these quasi-oscillations can be inferred from the periods of the ST-EOFs and ST-PCs (see Figs. 7 and 8, respectively; the time span is 120 months, or 10 years). MSSA confirms that the first two pairs, with cycles of one year and 6 months, respectively, are correlated with the monthly wave heights as their amplitude is largest at channel 2, which corresponds to the MWH. The other pairs have largest amplitude at channel 1; hence, they are all correlated with the NAO. Pairs 5–6, 7–8, 9–10 and 11–12 are linked to cycles of periods (in months) 3.92, 3.14, 8.62 and 2.96, respectively. Pair 13–14 is associated with two cycles: of 5.4 months and 2.98 months. From the ST-PC of Pair 13–14 one can see this pair is associated with a semi-annual cycle which could be associated with NAO or MWH as the amplitudes at both channels have similar amplitudes. It is noteworthy that no collective inter-annual patterns seem to exist, even if such patterns have an important influence locally.

## Discussion

### 4.1 Sensitivity to Changes in Window Length for SSA

The results have shown some limitations of SSA in unequivocally identifying patterns with periodicities close to the size of the window length. Hence, we tested the robustness of the patterns to small window length changes.

#### 4.1.1 Transect 58

A window length of 4 years was chosen for the detrending analysis at this transect. However, the robustness of the results to small changes in window length was tested by varying the window between 3.3 and 6.6 years. This is so that the window length span includes the sandbar life cycles observed North and South of the pier. The extrema had approximately the same values for all of the window lengths considered, with some small deviations of their location along the transect depending on window length. The main difference observed occurred in the region of small variance, i.e. between 180 m and 220 m offshore, where the trend signal did not capture any of the extrema when *M* = 3.3 years. However, window lengths between 4 and 6.6 years extracted trends that have similar characteristics.

The LPOs and SPOs within the two parts of the original signal thus obtained were then identified with SSA at a window length of 9 years and a window length of 8.12 years. Again, the dominant patterns were found to be robust to changes in window length, but the 9-year window length captured some of the fluctuations better.

#### 4.1.2 Transect 190

Since the window length of 4 years was best at transect 58, detrending was performed initially at this window length. The trends shown in Fig. 2E are at this window length for most of the transect except between 130 m and 170 m offshore. In this region, the 4-year window did not resolve the travelling patterns adequately; thus, it was necessary to experiment with slight changes to window length to obtain a coherent picture. The amount of variance resolved by the patterns, however, is the same as that with a 4-year window. The characteristics of the travelling patterns are therefore robust because these slight changes do not introduce more information. It seems that the sensitivity in window length in this region is due to the change in type of behaviour between the region closer to the shore and the patterns travelling seawards between 140 m and 270 m (see the patterns developing from year 15 onwards in Fig. 2E, for instance).

### 4.2 Comparison with Previous Duck Studies

Our results compare well with previous wavelet analyses focusing on temporal scales of variability at transect 62 [33]. Transect 62 lies only around 100 m south of transect 58, so they are sufficiently close for variations in underlying oscillations to be small. We shall therefore use our results for comparison.

At transect 62 it was found that, between 100 m and 190 m offshore, most of the variance corresponded to periodicities between 8 and 12.8 months. This agrees well with the yearly pattern found at transect 58. However, our trend analysis has highlighted that LPOs are also present in this region, with periodicities ranging between 3 and 12 years depending on the location. SSA at 260 m led to three dominant quasi-oscillations of periods: 4.5–5.9 years; 1.6–2 years; and 3.6–4.8 years. This agrees relatively well with the patterns in transect 62 at *x* = 260 m, where 25.95% of the variance corresponded to periodicities between 1.3 and 1.8 years. This is linked to the 1.6–2-year patterns identified at Transect 58. It is noteworthy that at *x* = 410 m, the pattern with a period of 6−8 years found in transect 62 was not observed in transect 58. Therefore, the alongshore homogeneity assumption at this position breaks to some degree. This does not significantly affect the analysis presented here since it is not based on this assumption.

The large variability of temporal scales identified within the dynamics is a consequence of the extremely non-stationary behaviour of beach profiles at Duck. Non-stationarity of patterns, observed in transect 62 with the wavelet method [32], [33], is also evident with SSA in transect 58. It has been pointed out that SSA and wavelets can both identify non-stationary behaviour in time series, contrary to more standard time series analysis methods [52]. Also, although wavelets have been the method of choice for intermittent time series analysis, a multi-scale SSA may be produced by using a moving window length and the eigenvectors of the resulting lag-correlation matrix are equivalent to data-adaptive wavelets. While wavelet analysis may appear conceptually simple, it is not without difficulties. For instance, the Heisenberg uncertainty principle implies that the optimizing wavelet needs to satisfy a trade-off between localisation in time and in frequency [52], [53]. It would be interesting to analyse correlations between the bed-level behaviour with cross-wavelets between the bed-level variations and the suggested potential forcings, at individual locations. This would permit the identification of the relative phases between the two time series, which is not straightforward with SSA. It would also provide an additional measure of the correlation between the bed-levels and the phenomena time series at given locations.

Sequential beach change studies of Duck focusing on the period between August 1981 and July 1984, i.e., from *t* = 0 to *t* = 3 yrs [11], and the period between October 1986 and September 1991, i.e., from *t* = 5.35 to 10.17 yrs [54], can be compared to the results presented here. In both studies the shoreline moved on and offshore, always lying between *x* = 100 m and *x* = 140 m. This agrees well with the first spatial region identified with SSA at both window lengths, which implies that these quasi-oscillatory patterns are linked to those of the shoreline.

There is also good agreement between the three studies in identifying the most dynamic region. In this study it is found, for instance, that between 130 m and 350 m several quasi-periodic cycles may dominate the dynamics (see panels B and C as well as panels E and F in Fig. 2). In the sequential beach models this was also the most dynamic region in terms of number of bars and in terms of bar motion, with the inner bars moving on- and offshore between 130 m and 330 m, and the outer bars between 200 m and 450 m. Moreover, at transects 62 and 188 the inner bars had significant intra-annual variations and remained generally shorewards of 200 m–210 m offshore [11]. In this study it is also found that several intra-annual patterns exist shorewards of 200 m–210 m, as shown in panels C and F of Fig. 2.

Finally, note that patterns with longer term periodicities, such as the 20-year periodicity associated with storm groups [55], cannot be seen in a study with a window length of 9 years. We would need to extend the analysis and use a window length of at least 20 years to be able to identify them.

### 4.3 Correlations of NAO, MWH and MWL Patterns with Bathymetric Patterns Along Transects 58 and 190

We hypothesise that the semi-annual and the annual cycles found in the bathymetric profiles are very likely to be induced by the monthly averaged wave climate. This is corroborated by the MEOF and the MSSA studies discussed in Sect. 4.4 and confirms extensive work by previous authors [8], [11], [17], [18], who have identified wave climate as one of the dominant forcing mechanisms of near-shore dynamics.

Some of the patterns within the MWL are similar to those embedded within the bathymetry at some locations. Note that in studies on mudflats the neap-spring tide cycles and the seasonal cycles were correlated with cycles within tidal and wave conditions, while bathymetric long-term patterns were strongly correlated with the NAO [56]. Considering the inter-annual MWL patterns that we identified at Duck, MWL may be influencing such long-term patterns in the bathymetry, and not just tidal or seasonal patterns. However, the mudflats study and other investigations on the influence of climatic patterns on bed-level variations, would suggest that NAO may be influencing bed-level variations at Duck [36], [56], [57].

Some of the periodicities within the bed-level data coincide with the observed processes, but not with others. One should not be seeking a physical explanation for all the statistics, as noise produced by transverse bars or bar splitting, for example, may cause some variations in the observations [56]. However, conceptual models describing the sediment transport and hydrodynamic processes, expected to result in the observed patterns, need to be based on links between the patterns so that causalities can be identified. For instance, at Lubiatowo a weak correlation between NAO and the wave climate has been observed [36]. On the other hand, accretion patterns in the Southern end of Narrabeen Beach, New South Wales, Australia, have been linked to negative Southern Oscillation Index (SOI) phases [57]. Indeed, these accretion patterns were linked to a reduced number of tropical cyclones and to East Coast lows in the Australian East Coast, which pushed the storms further South, leading to a reduction of their effects on the side of pocket beaches sheltered by headlands. In the North Atlantic, the NAO has been shown to produce variations in sea level over decadal timescales [58]. At short-to-medium timescales, NAO index phases are likely to be linked to changes in the lows and highs of sea level pressure. This has an effect on the direction and intensity of the storms, in analogy with the observed effects of the SOI in the South Pacific Ocean. Most research to date has identified links between climatic indexes, erosion and accretion patterns, but it is more difficult to assess their effects on the dynamics of NOM systems. At Duck, the pier may also be introducing patterns similar to those observed on sheltered beaches depending on the direction of the incoming waves. Additional research, looking at NAO index phases, would be necessary to propose any conceptual model on how NAO may be influencing the dynamics at Duck. This is beyond the scope of the current investigation which is only concerned with quantification of cyclic patterns and is left for future studies. We did perform spatio-temporal correlation analyses using MEOF and MSSA in order to further analyse whether the potential forcings discussed in this section are correlated with seabed level measurements throughout the bathymetry. Which collective patterns of behaviour they may be correlated with was also examined.

### 4.4 Spatial and Spatio-temporal Correlations between NAO, MWH and MWL and Overall Bathymetry

As shown in Sect. 3.3, the MEOF has permitted the isolation of the effects of the different potential forcings on the bathymetry. This is because each potential forcing is most strongly correlated with one of the first three EOFs: the NAO with EOF1; the monthly wave heights (MWH) with EOF2; and the monthly mean water level (MWL) with EOF3.

Figures 6A–6C permit the identification of regions where each of the potential forcings is more strongly correlated with seabed evolution, simply by locating the regions where the correlations are highly positive or highly negative. Note that the region where the analysis was performed extends from 80 m to 520 m offshore, rather than between 100 m and 490 m, as was the case in the previous sections. As previously explained, this was done to capture the behaviour around the shoreline and the behaviour offshore more fully.

To simplify the discussion we define near-shore (80–200 m), mid-shore (200–270 m) and far-shore (>370 m) regions. Since the first EOF captures the largest proportion of the variance and is linked to the NAO, the NAO appears to be more strongly correlated with the seabed than waves at the time and space scales under consideration. Fig. 6A shows the correlation distribution between the seabed and NAO and highlights the complex interactions between them; the regions of positive and negative correlation are clearly visible in the plot. It shows the correlations are either very weak (with |*r*|<0.1) or weak (with 0.1<|*r*|<0.3). Weak correlations occur in the following locations: 1) North of the pier, in the near-shore region, 600 m–1100 m alongshore; 2) South of the pier, in the far-shore region, between −100 m and 100 m alongshore; 3) all along the shore between near- and mid-shore regions. However, even though the correlation is low, it is found that the correlation coefficient contours give valuable information and the locations of extrema (either positive or negative) are more important that the magnitude of the extrema. An analysis of panel 6A close to the pier shows that there are stronger correlations between NAO and the seabed in the mid- and far-shore regions, than at other cross-shore locations. Moving northward from the pier these regions, while remaining distinct, move closer to the shore. Thus, at transect 58, the largest negative correlations occur in the near- and far-shore regions; and two regions of positive correlations appear in the far-shore region. Southwards of the pier, the region of negative correlation is in the near-shore and a region of positive correlations appears in the far-shore. Note that although the magnitude of the correlations is low, the variance resolved by the EOF associated with the NAO is large as the correlation coefficient is linked to the spatial EOFs of the MEOF covariance matrix (see Table 3). The variance is a relative measure indicating that, along this direction, correlations with other phenomena will be much weaker.

The SSA deduced correlation between regions where NAO and the bathymetry are more likely to be correlated (or anti-correlated), relative to other seabed locations, appears to be consistent at transects 58 and 190. This holds even if, in absolute terms, the correlations everywhere are weak. For instance, Table 2 shows that, for transect 58, regions seawards of 150 m have one underlying cycle at a periodicity found within the NAO signal; the NAO has three inter-annual underlying cycles with periods of around 2, 5 and 6–8 years, respectively. If, for instance, we choose the 250 m offshore location, we see that the period of 5 yrs is contained within the bathymetric pattern with period 5.4±0.9 yrs; at 480 m and 490 m, two of the dominant bathymetric cycles identified have periods that are close to two dominant patterns within NAO, namely the 2-year and the 6–8-year cycles. For transect 190, patterns of the same periodicity as those of the NAO were found both from 120 m–200 m, and beyond 450 m offshore. A possible physical mechanism for the NAO to influence the bathymetry indirectly is through the thin layer of water in the near-shore, producing cyclic patterns of behaviour in the bathymetry, with periodicities close to its own underlying cyclic patterns. This is a highly speculative argument based on scales: the NAO patterns have a spatial scale of *O*(10^6^ m), but the water depth in the near-shore is only *O*(m).

The MWH is the dominant potential forcing in EOF2 and the distribution of correlations between MWH and the seabed is shown in panel 6B. Again, the regions around positive- and negative-correlation extrema indicate regions where the MWH is more likely to be correlated with the seabed oscillations. There is agreement between the regions identified here and those where the waves may have an important effect. e.g., in the swash zone, between 80 m and 90 m offshore, where there are positive correlations. We know from the SSA that the wave height has a yearly pattern and that such a pattern is also present in the near- and mid-shore at transect 58 and all along the transect at transect 190. As discussed above, Fig. 6B seems to indicate that the correlation between the seabed, and the waves, near the pier, and close to the shoreline is stronger than at other bathymetric locations. This is evidenced by the region of negative correlations occurring in the near and mid-shore and between 0 m and 800 m alongshore, approximately. As we approach transect 190, there appears to be a correlation minimum in the mid-shore and a correlation maximum in the far-shore, even though the correlations are weak overall. At transect 58, there appears to be more variation in the correlation values, possibly indicating more variability in the interaction between the waves and the seafloor along this transect than along transect 190.

Finally, panel 6C shows the distribution of correlations between the seabed and the MWL. The figure shows regions of moderate correlation and the distribution of correlation coefficients is symmetric around the pier. The plots suggest the greatest correlation with the MWL are in the near- and the far-shore along transects 58 and 190. This is relatively consistent with the locations where a 1.4–2.2 yearly cycle was found (see Table 2), thus with previous wavelet findings [19]. This work not only confirms the presence of those bathymetric patterns but also suggests that the physical mechanism influencing them is one which, at least partially, contributes to changes in water level variations. These could be any of the storm surge components.

**Figure 9:**
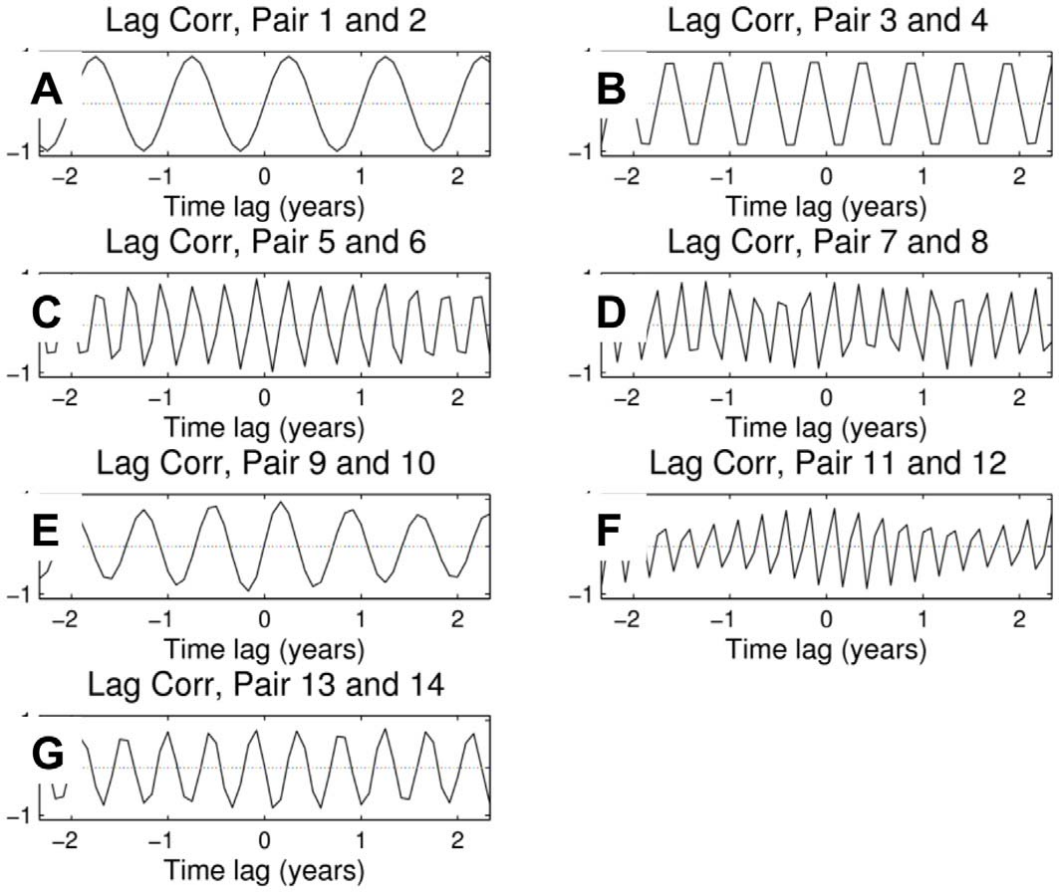
Lagged correlations for all ST-PC pairs.

The remainder of this section focuses on the MSSA results. The MSSA methodology is described in Appendix S1 where the ST-EOFs (shown in Fig. 7), St-PCs (shown in Fig. 8) and lagged correlations between all ST-PC pairs (shown in Fig. 9) are discussed in detail. As presented in Sect. 3.3, no inter-annual coherent, spatio-temporal patterns are found when all of the bathymetric measurements, and the three potential forcings, are analysed together, even when these patterns are clearly identified locally. This is an important difference with the SSA/MEOF analyses in which the local bathymetric patterns could be attributed to any of the three forcings considered, depending on the characteristics of the patterns and the region under consideration. However, the absence of coherent inter-annual, spatio-temporal patterns could mean that the mechanism forcing such behaviour acts locally, and not globally, at these temporal scales.

For studies including monthly responses, the results in Sect. 3.3 show that the two most important collective patterns - the yearly and the semi-yearly cycle - are both correlated with the monthly wave heights. This agrees well with the SSA results summarized in Table 2, showing the ubiquitous presence of the annual cycle all along transects 58 and 190. Finally, except for the semi-annual pattern, all of the intra-annual collective patterns identified are correlated with the NAO.

### 4.5 Summary and Conclusions

Local and collective bathymetric quasi-periodic patterns of oscillation were identified from monthly profile surveys spanning 26 years, from July 1981 to January 2006, at the USACE field research facility in Duck, North Carolina. The local analyses were based mostly on the outermost transects of the surveyed domain, but the collective patterns were computed using all surveyed bathymetric profiles, including those in the vicinity of the pier. Correlations with three potential forcings, namely the monthly wave heights (MWH), monthly mean water levels (MWL) - which include the storm surges as well as the tide - and the large scale atmospheric index, the North Atlantic Oscillation (NAO), were discussed.

The local seabed patterns and their spatial correlations with NAO, MWH and MWL were first analysed using SSA. This showed highly non-stationary behaviour of the bed-levels along transects 58 and 190. It was shown that: 1) a quasi-yearly cycle was embedded at most bathymetric locations, most likely correlated with the quasi-yearly pattern within the monthly averaged wave climate; 2) the local behaviour is highly non-stationary as observed by previous studies; 3) patterns with periodicities within the range of 1.4–2.2 years were found at some seabed locations and within the MWL, providing an explanation for the potential origin of such patterns; 4) several inter-annual patterns were identified, some of which are likely to be correlated with the NAO. Some statistics may be attributed to noise. As in other studies at Duck, differences in the behaviour North and South of the pier were observed.

Linear correlation coefficient contours were computed throughout all bathymetric surveys. The regions at the North and South boundaries of the domain, where the magnitude of the correlation coefficient was largest relative to other locations along the transect, seem to agree broadly with those identified with SSA. This is despite the correlation coefficient giving a measure of linear correlation while SSA is a non-linear measure; linear correlation analysis cannot characterise the non-linear quasi-oscillations. It was found that linear correlations with the NAO and MWH were weak to very weak. For the MWL, the correlation contours were symmetric North and South of the pier and there was a region between 100 m and 200 m offshore of moderate correlation between bed-levels and MWL for all of the surveys.

The last component of the analysis consisted of the identification of spatio-temporal oscillations and their non-linear correlation with NAO, MWH and MWL. This indicated that there are no patterns that are coherent throughout the bathymetry at yearly timescales; there are a few at monthly scales which are correlated with the NAO. This is consistent with the highly localised nature of the inter-annual, quasi-oscillatory patterns that were observed with SSA. This study is a preliminary step towards, for instance, an understanding of the potential effects of increased storminess (due to climate change) on bathymetric evolution, or attempts to model near-shore, long-term morphodynamics.

## Supporting Information

Appendix S1 **Description of Methods.**
(DOCX)
