# Supplementary material for: Spectral Quantification of Nonlinear Behaviour of the Nearshore Seabed and Correlations with Potential Forcings at Duck, N.C., U.S.A
Source: PLoS One. 2012 Jun 26;7(6):e39196. doi: 10.1371/journal.pone.0039196 (PMC3383760; doi:10.1371/journal.pone.0039196)
Supplement: Appendix S1 — Description of Methods. (DOCX) [file pone.0039196.s001.docx]

## APPENDIX S1: Description of methods

### S1.1 SSA and SDA

The fundamental theory of SSA was developed in the 1980s for dynamical systems and discrete times series analysis, and here a summary of the methodology [21, 59, 60]. The following summarises the SSA methodology. Suppose the bed level variable *z*(*t*), sampled at times *t= iτ_s_*, *i*=1,2,…,*N* (*N* being the length of the time series and *τ_s_ *the sampling interval), at a given cross-shore and long-shore location (*x*,*y*), characterises the seafloor dynamical system. First, the noise part of the bed level time series may be identified by computing the statistical dimension, *S*, which is found by constructing a secondary, *M*−dimensional sequence, *Z_n_*=(*z_n_,z_n+1_,...,z_n+M-1_*), for *n*=1,2,…,*N*−*M*+1, where *M*<*N* is the window length and *z_n_=z(nτ_s_)*. Time scales of the dynamics that can be reconstructed from this time series are between *τ_s_* and *Mτ_s_*, the window time span. Then, the eigenvalue problem

$C\mathbf{e}^{(k)}=\text{}_{k}\mathbf{e}^{(k)}$ (1)

must be solved, where *C* is the covariance matrix of the sequence *Z_n_* and *λ_k_* are the eigenvalues associated with the eigenvector **e***^(k)^*. The square roots of *λ_k_* are called the singular values, with their set being called the *singular spectrum*. The largest singular values relate to the directions that resolve most of the variance, with the rest being a representation of the noise component and appearing as an approximately flat floor in a plot of singular value *vs.* singular value rank (with the singular values ordered from largest to smallest). The dimension *S*, then, will be given by the number of singular values above this floor.

Now, each singular value has an associated principal component (PC), defined as

$\text{}_{k}\left( i\text{}_{s} \right)=\sum_{j=1}^{M} z\left\{ \left( i+j-1 \right)\text{}_{s} \right\}\mathbf{e}_{j}^{(k)}$ (2)

Once the PCs have been identified, the part of the original time series related to that PC, or to a combination of *K* PCs, may be obtained by computing

$RC\left( i\text{}_{s} \right)=\frac{1}{M_{i}}\sum_{k\in K} \sum_{j=L_{i}}^{U_{i}} \text{}_{k}\left\{ \left( i-j+1 \right)\text{}_{s} \right\}\mathbf{e}_{j}^{(k)}$ (3)

where RC, the *reconstructed component*, is the part of the original signal related to the PC combination. The values of the renormalisation factor, *M_i_*, and the lower and upper bound, *L_i_* and *U_i_*, respectively, depend on the time at which the reconstruction is being evaluated.

The frequencies that can be resolved with SSA depend on the choice of window length, *M*, which is a free parameter of the method. However, the patterns extracted should be robust to small changes in window length. Here the depth time series were divided into a trend and a detrended signal. The trend generally contains a part found by linear regression of the data, as well as the RC corresponding to the first few eigenvectors of the SSA at a given window length. These first few eigenvectors generally contain all oscillations within the original time series that have periods above *M*, which in this case was taken as 4 years. From the trend, thus computed, long-period oscillations (LPOs) were extracted, while short-period oscillations (SPOs) may be characterised from the detrended signal. Cycles of 3‒5 years may fall in either signal because of the window length selected for the detrending analysis (this was discussed in some detail in Sects. 2.2, 3.2 and 4.1).

In natural systems, there is the additional problem of red noise (which is noise composed of many random frequencies but, in contrast to white noise where all frequencies have equal intensity, the intensity of the lower frequencies is stronger) that can be removed from the data by using either Monte Carlo or chi-squared tests. In this work a chi-squared test was applied on 100 red-noise surrogates; these surrogates were projected onto the SSA eigenvector basis, and the distribution of projections was approximated as chi-squared with 3*M*/*N* degrees of freedom. Then, projections that fell outside of a percentile range of 2.5‒97.5% were identified as being part of the signal; thus, these could be dissociated from the red noise in the time series.

Spectral density analysis (SDA) is a well established technique to analyse oscillatory patterns in time series. The spectral density was computed using Welch’s periodogram method and the Blackman-Tukey correlogram approach [61, 62]. The former consists of dividing the input signal into short segments and computing a modified periodogram for each segment, thus leading to a set of periodograms which are finally averaged to obtain an estimate of the spectral density function. In the latter a windowed Fast Fourier Transform of the autocorrelation function is equated to the spectral density.

It is well known that the spectral density is a measure of the energy contained in the system. By analysing the spectral density it is possible to characterise how the total energy is distributed and, in particular, to find the frequencies that contribute the most to it.

### S1.2 MEOF/Correlation analysis

Multivariate empirical orthogonal functions can be used to study the relationship between different physical variables that are sampled at the same set of times. It can be thought of as a prefiltering step for MSSA/MEEOF analysis: instead of performing MSSA directly on the data, one first finds the MEOFs and uses time-lagged copies of the corresponding PCs in the MSSA, in order to reduce the dimension of the covariance matrix.

We construct a matrix *B* whose elements $B_{ij}=x_{i}^{(j)}$are the values of the *j*th variable sampled at time *iτ_s_*, where *i*=1,2,...,*N* and *j*=1,2,...,*L*, where each vector ***x***^(j)^ contains the full time series for a particular variable. The full set of variables might include samples of the same physical variable at different locations and/or of different physical variables at the same location.

We next subtract from each element of $x_{i}^{(j)}$the mean value of all its elements, to form a matrix *F* consisting of columns $\mathbf{y}^{(j)}$with zero mean:

$${F_{ij}=y}_{i}^{(j)}{=x}_{i}^{(j)}-\frac{1}{N}\sum_{i=1}^{N} x_{i}^{(j)}$$

Since the columns of *F* represent different physical variables, we renormalise them so that the results of the analysis are not influenced by the units in which we choose to measure them [35]. Our data consists of measurements of the bathymetry at 1125 separate locations over Duck beach (covering the area between *y*=−91 to 1097 m along the shore and between *x*=80 to 520 m offshore), together with measurements of the monthly mean water levels (MWL) and monthly wave heights (MWH) at a wave gauge located near Duck beach, and a measurement of the North Atlantic Oscillation (NAO) index spatially averaged over the whole surveyed area, the NAO index (or NAO in short) being the pressure difference between Iceland and the Azores. Since all of these forcings were simultaneously available for only the last 19 years of bathymetric surveys, we reduced the analysis to this duration. We considered two possible choices for the renormalisation factor: the range of each variable or its standard deviation. We decided to use the range because we were interested in understanding the effect of each possible forcing independently, and using the range separated these out in the calculated MEOFs to a greater degree than using the standard deviation. Finally, since we have 1125 pieces of data for the bathymetry at each time *iτ_s_* and only one piece for each possible forcing, we divide the bathymetry measurements by 1125 so that the effect of the potential forcings is comparable with that of the spatial variations in the bathymetry. From now on we consider *F* to have been renormalised in this manner.

Next, the EOF decomposition is performed. EOF analysis is equivalent to Principal Component Analysis (PCA), and to singular value decomposition (SVD). SVD of *F*permits the identification of bathymetric locations where the bathymetry may be strongly correlated with each of the potential forcings. Such decomposition is of the form

$$F=USV^{T}$$

where the columns of *V* are the EOFs of the SVD, or the spatial eigenvectors, the columns of *U* are the PCs, or the temporal eigenvectors and *S* is the diagonal matrix of eigenvalues; the ratio of each eigenvalue divided by the sum of all of the eigenvalues gives the fraction of the total variance explained by each eigenvector. The EOFs represent the stationary patterns in the bathymetry and potential forcings that explain most of the observed variability, with the greatest variance being resolved by the EOF corresponding to the largest eigenvalue, and so on. The PCs provide the variation over time of the sign and amplitude of the associated EOF.

Each spatial eigenvector giving a direction in which each potential forcing is dominant gives an excellent proxy of the relative correlation of that phenomenon with that particular spatial mode of the bathymetry. In fact, computing the correlations directly from the data and comparing the correlation distributions to the spatial EOF contours would show that correlation contours and EOF contours are essentially equivalent. This is because of the dominance of the phenomena along well defined EOF directions. Since linear correlations are conceptually more straightforward than EOF distributions, we present and discuss the correlation plots instead of the EOF distributions. However, we use MEOF as a filter for the MSSA.

### S1.3 MEEOF/MSSA

Multivariate Extended Empirical Orthogonal Functions (MEEOF) is the name given to Multichannel Singular Spectrum Analysis (MSSA) when several different physical variables are included in the analysis. Such a technique has been useful, in particular in the analysis of the behaviour of climatic phenomena, with the aim of extracting modulated oscillations from the coloured noise commonly present in natural systems [21, 35]. Its use has focused on accurate identification and characterisation of the system’s temporal and spectral properties. A short description of MSSA is presented for the reader interested in the methodology and its capabilities.

Suppose we have the dataset, ${\{y}_{i}^{(j)};i=1,\ldots,N,j=1,\ldots L$}, discussed in the previous section. Several techniques to produce a matrix of time-lagged vectors may be proposed. Matrices $\tilde{Y}^{(j)}$ are formed by setting the channel to *j*,$1\leq j\leq L$, sliding a vector of length *M* down from *i*=1 to *i*=*N*'≡*N*−*M*+1 and ordering the resulting vectors from left to right as the columns of $\tilde{Y}^{(j)}$**.** Thus each $\tilde{Y}^{(j)}$ has size *M*×*N*'. This leads to a *trajectory matrix*,

$\tilde{Y}^{(j)}=\left( \begin{aligned} {\tilde{\mathbf{Y}}}^{\left( 1 \right)} \\ \vdots\\ {\tilde{\mathbf{Y}}}^{(L)} \end{aligned} \right)$(4)

of size *ML*×*N*'.

A singular value decomposition of $\tilde{Y}$ may then be performed, such that

$\tilde{Y}^{(j)}=P_{\tilde{Y}}\text{}_{\tilde{Y}}^{1/2}E_{\tilde{Y}}^{T}$ (5)

Where $P_{\tilde{Y}}$ consists of the temporal principal components (T-PCs). As equation (5) implies, these are the left-singular vectors of$\tilde{Y}$. The matrix $E_{\tilde{Y}}$ consists of the spatio-temporal empirical orthogonal functions (ST-EOFs) corresponding to the right-singular vectors of $\tilde{Y}$. Finally, $\text{}_{\tilde{Y}}$is the diagonal matrix of variances associated with these orthogonal bases.

The bases $P_{\tilde{Y}}$ and $E_{\tilde{Y}}$ are the eigenvectors of the covariance matrices

$C_{\tilde{Y}}^{E}=\frac{1}{ML}\tilde{Y}\tilde{Y}^{T}\text{and }C_{\tilde{Y}}^{E}=\frac{1}{N^{'}}\tilde{Y}^{T}\tilde{Y}$ (6)

respectively. However, it is only for the smallest of $C_{\tilde{Y}}^{P}$ and $C_{\tilde{Y}}^{E}$ that either $P_{\tilde{Y}}$ or $E_{\tilde{Y}}$ is full-rank [63, 64]. Therefore, the choice of *M* determines which of the eigenbases is to be analysed. However, the method of construction of $\tilde{Y}$ implies that the column vectors of $\tilde{Y}^{T}$are time-lagged copies at a given channel, so it is convenient to choose *N*' sufficiently small to satisfy *N*'<*ML*, so that the full rank matrix is $C_{\tilde{Y}}^{E}$.

As mentioned in Sect. , rather than performing the MSSA on the data and having to resolve a very large covariance problem, it is better to perform a data reduction procedure by prefiltering the original data with the MEOF and applying the MSSA to the resulting EOFs. In this way, the most important information is compressed into a small number of variables and the analysis of the coherent patterns is simplified significantly [51]. In this case the first three eigenvalues of the MEOF resolve most of the variance and also are the ones in the directions of the three potential forcings considered, so MSSA can be applied to these three eigenfunctions rather than to the data.

The first 14 MSSA components of the filtered system – that is, the system consisting of the first 3 MEOF PCs as input channels, were analysed. Each of these components has its corresponding eigenvalue, spatio-temporal PC (ST-PC) and spatio-temporal EOF (ST-EOF). A window length of *M*=120, equivalent to *N*'=9 years, was chosen. The method consists of finding pairs of consecutive eigenvalues (*k*, *k*+1) for which [51]:

• the two eigenvalues are nearly equal, that is, they are both within the margins of error identified by North’s rule of thumb [65],

• the two corresponding time sequences described by the ST-PCs are nearly periodic, with the same period and in quadrature,

• the associated ST-EOFs are in quadrature and

• the lag correlation between ST-EOFs of order *k* and *k*+1 itself shows oscillatory behaviour [66].

Pairs that pass all of these tests are associated with quasi-oscillations with periods equal to the period of the ST-PCs - which is also the period of the ST-EOFs - in the embedding space, here spanning 121 months.

The first test was to inspect pairs of consecutive eigenvalues *λ_k_* and *λ_k_*_+1_to see if they satisfied North’s rule of thumb. It was found that eigenvalue *λ_k_*_+1_ is between *λ_k_* - *λ_k_*$\sqrt{1/N}$, and *λ_k_* + *λ_k_*$\sqrt{1/N}$, with *N*=228 months being the sampling duration, for all 7 pairs considered, so all satisfy the first criterion. Then plots of the ST-PCs for the 14 MSSA components at channel 1 showed that all pairs considered are quasi-periodic with same period and in phase quadrature; thus, they also satisfy the second criterion. This led to a check of the quadrature criterion for the ST-EOFs associated with each ST-PC pair. Since the ST-EOFs are the same for all of the PCA channels in the prefiltering, only the results for channel 1 are necessary. The ST-EOF plots are shown in Fig. 7 for the first 6 pairs, with the darker solid line corresponding to the eigenvector with smaller index. The ST-PCs are shown in Fig. 8. All of the pairs (including pair 13-14, not shown in Fig. 7 but shown in Figs. 8 and 9) pass the test. The final test consists of analysing the lagged correlation plots for the pairs which have satisfied all previous tests. The plots, shown in Fig. 9, should depict oscillatory functions. Again, all pairs pass this test.


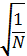


References

[59] Broomhead DS, King GP (1986) Extracting qualitative dynamics from experimental data. Physica D 20: 217–236.

[60] Ghil M, Allen MR, Dettinger MD, Ide K, Kondrashov D, et al. (2002) Advanced spectral methods for climatic time series. Rev Geophys 40 (1): **1**–1–**1**–41.

[61] Welch PD (1967) The use of fast Fourier transform for the estimation of power spectra: a method based on time averaging over short, modified periodograms. IEEE Trans Audio Electroac 15 (2): 70–73.

[62] Blackman RB, Tukey JW (1958) The measurement of power spectra from the point of view of communication engineering. New York: Dover Publications Inc. 208 p.

[63] Allen MR, Robertson AW (1996) Distinguishing modulated oscillations from coloured noise in multivariate datasets. Clim Dyn 12(11): 775–784.

[64] Robertson, AW (1996) Interdecadal variability over the North Pacific in a multi-century climate simulation. Clim Dyn 12: 227–241.

[65] North GR, Bell TL, Cahalan RF, Moeng FJ (1982) Sampling errors in the estimation of empirical orthogonal functions. Mon Wea Rev 110: 699–706.

[66] Ghil M, Mo KC (1991) Intraseasonal oscillations in the global atmosphere. Part I: Northern Hemisphere and tropics. J Atmos Sci 48: 752–779.
